# Why mismatch negativity continues to hold potential in probing altered brain function in schizophrenia

**DOI:** 10.1002/pcn5.144

**Published:** 2023-09-24

**Authors:** Juanita Todd, Dean Salisbury, Patricia T. Michie

**Affiliations:** ^1^ School of Psychological Sciences University of Newcastle Newcastle New South Wales Australia; ^2^ Department of Psychiatry University of Pittsburgh School of Medicine Pittsburgh Pennsylvania USA

**Keywords:** auditory, biomarker, mismatch negativity (MMN), NMDA, schizophrenia

## Abstract

The brain potential known as mismatch negativity (MMN) is one of the most studied indices of altered brain function in schizophrenia. This review looks at what has been learned about MMN in schizophrenia over the last three decades and why the level of interest and activity in this field of research remains strong. A diligent consideration of available evidence suggests that MMN can serve as a biomarker in schizophrenia, but perhaps not the kind of biomarker that early research supposed. This review concludes that MMN measurement is likely to be most useful as a monitoring and response biomarker enabling tracking of an underlying pathology and efficacy of interventions, respectively. The role of, and challenges presented by, pre‐clinical models is discussed as well as the merits of different methodologies that can be brought to bear in pursuing a deeper understanding of pathophysiology that might explain smaller MMN in schizophrenia.

A Web of Science search reveals over 733 papers on mismatch negativity (MMN) in schizophrenia since the early 1990s, averaging about 30 a year for the last 10 years (search title contains schizophrenia and topic contains mismatch negativity). There are more than 90 review papers with about four published per year for the last 20 years, including four meta‐analyses all supporting a replicable difference in MMN in schizophrenia.^1^, ^2^, ^3^, ^4^ These levels of output indirectly convey high levels of continued input into a field of research that has been active for over 30 years. With all this behind us, how close are we to being able to explain what smaller MMN reveals about the biological changes taking place in the brain in schizophrenia and how that knowledge might advance diagnosis, new treatment, and prevention? In this review, we explore the types of questions being asked of schizophrenia using MMN to highlight where a consensus has emerged but also to propose where and why open questions remain.

## WHAT PROCESS DOES MISMATCH NEGATIVITY (MMN) INDEX?

**Figure 1:**
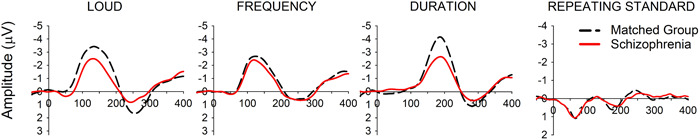
A group‐averaged deviant−standard difference waveform for loud (90 dB), frequency (1200 Hz) and duration (120 ms) deviant tones relative to common repeating standard tones (75 dB, 1000 Hz, 60 ms) for 33 persons with schizophrenia (red solid line) and 30 age‐matched controls (black broken line) at the frontocentral site *Fz*. The image is adapted from a published data set^6^ and features data from a traditional oddball paradigm in which the response to repeating standard sounds is very small and also typically very similar in schizophrenia and matched groups but the response to deviations from the standard, and therefore the resultant difference waves tends to differ between groups. In this particular study, the group differences were more pronounced for changes in sound duration and intensity/loudness than for frequency. The MMN component is evident between 100 and 200 ms for loud and frequency deviants and 150–250 ms for duration.

In the broadest sense, MMN indexes an automatic comparison process in which incoming sensory information is compared to a memory incorporating information about what is most probable in the current context.^5^ Most schizophrenia studies measure MMN using electroencephalography (EEG) to record responses to a structured sequence of sounds, although MMN can also be observed using other neuroimaging techniques, such as magnetoencephalography and functional magnetic resonance imaging. Using this methodology, MMN is visible as a noticeable difference in an event‐related potential to an unexpected or deviating event and is within about 100–250 ms from event onset (e.g., see Figure 1).^7^ Three necessary conditions to the elicitation of MMN include that the deviation must be sufficiently rare (generally *p* < 0.30), the deviation must be discernibly different from an established regularity (i.e., associated with a sufficiently unique neural representation), and an active contextual memory must be present. The latter is associated with some known temporal constraints in that MMN requires temporal proximity between sounds and an active context. For example, in simple traditional oddball sequences a probable repeating sound is occasionally interrupted by a rare physically deviant sound that elicits MMN unless the timing between sounds exceeds about 10 s.^8^ MMN also requires a recent preceding regularity; if there is a long break within a sequence (e.g., 30 s), a subsequent deviation will only elicit MMN if it occurs after an occurrence of the regular sound which is required to “reactivate” the prior context.^9^, ^10^, ^11^ MMN therefore indexes the response to improbable violations of an established, contextually relevant regularity. Importantly, MMN is observed in persons with schizophrenia, but the amplitude of this response is reduced.^6^ In traditional oddball sequences the response to repetitive sounds does not typically differ over the period of the MMN in schizophrenia but the response to deviations is significantly smaller (e.g., see Figure 1).

Functionally, MMN is reliant on automatic perceptual inference where what has been learned from sensory input patterns is used to infer the likely next state.^12^, ^13^ This anticipation is the attributed cause of progressively smaller responses to highly predictable sounds as a function of consecutive repeats^14^ and periods of pattern stability^15^ with the attributed consequence being efficiency in limiting the degree to which cognitive resources are drawn to what is already well known (can be predicted) about the environment.^16^ However, the presence of an active internal predictive model serves the concurrent function of signaling change via large MMN responses which can trigger an orienting of resources and attention and, potentially, the remodeling of the environment if the mismatches to predictions continue. Although most widely studied using auditory modality, MMN has been elicited in other sensory modalities, such as visual and tactile and unexpected input or the absence of expected input.^12^, ^17^ In this way, MMN provides an assessment of the integrity of learning that services a fundamental monitoring process that automatically filters the relative information value of sensory events.

MMN amplitude has been measured in schizophrenia to explore a wide variety of questions that have primarily centered around what it might teach us about the pathophysiology of the illness. MMN was first explored in schizophrenia within the context of studying cognitive processes, such as sensory memory, selective attention and the gating of information based on relevance.^18^, ^19^, ^20^, ^21^, ^22^ However, the ease of measuring MMN and the replicability of smaller MMN amplitude have contributed to interest in how it might relate to other observed brain changes. While theories put forward to explain the etiology of schizophrenia are many and varied, that most closely tied to the process underlying MMN is the glutamate hypothesis^23^ as it pertains to altered synaptic plasticity implicating disruption to *n*‐methyl‐d‐aspartate (NMDA) glutamate receptors, the excitatory–inhibitory balance in neurotransmission, and potential consequences for brain structure and connectivity.^24^, ^25^, ^26^, ^27^, ^28^, ^29^, ^30^, ^31^ Indeed, MMN has also been promoted as an exemplar of the synaptic plasticity involved in the fundamental role of “error‐signaling” in brain function generally^16^, ^32^ and altered inferential processing in schizophrenia specifically.^33^, ^34^, ^35^, ^36^, ^37^ While this paper does not offer an extensive review of these findings, attention is drawn to some of the most promising associations in the sections below. This review will cover the potential for MMN as a biomarker, the ways in which this potential is being exploited in pre‐clinical research and where some of the open questions remain (Figure 1).

## WHAT KIND OF BIOMARKER IS A REDUCTION IN MISMATCH NEGATIVITY?

There is considerable interest in using MMN as a biomarker to better understand schizophrenia^38^ but before exploring this potential in detail, it is necessary to appreciate that biomarkers come in many forms and functions and can be classified differently. A biomarker can be one of several types within a broader class of biological measures that index some aspect of pathology.^39^ Perhaps the most often assumed biomarker is one that indicates disease presence, a diagnostic biomarker. For example, elevated prostate‐specific antigen (PSA) levels are biomarkers for the detection of prostate cancer. Currently, there are no diagnostic biomarkers for the major mental illnesses, including schizophrenia. However, there are other types of biomarkers, and one measure may serve in several of these roles. In the United States, the National Institutes of Health (NIH) and the Food and Drug Administration (FDA) developed joint definitions of different biomarkers for research, medical, and interventional use. These comprise *diagnostic biomarkers* (e.g., elevated PSA for prostate cancer); *monitoring biomarkers* to track changes of the underlying pathology (e.g., reduced low‐density lipoproteins for cholesterol treatment); *response biomarkers* that are directly affected by interventions (e.g., changes in glucose for experimental diabetes‐related interventions); *safety biomarkers*, which assess possible side‐effects of some intervention (e.g., blood neutrophil counts for possible agranulocytosis with clozapine treatment); *predictive biomarkers* that assess the likelihood of positive or adverse effects from a specific intervention, and are useful for selection of the best intervention (e.g., specific treatment for HER2 breast cancer); *prognostic biomarkers* that indicate which individuals in whom a disease might occur, might recur, and aspects of the disease course (e.g., tumor size or metastasis in cancer); and *susceptibility/risk biomarkers* that indicate which individuals might transition from a healthy state to a pathological state (e.g., C‐reactive protein and risk for stroke).

A biomarker can be, but is not necessarily, an intermediate endophenotype. Intermediate endophenotypes are biological measures of complex physical traits, whether static or dynamic, that are linked to underlying genotypes that may, in turn, confer risk for certain disease states. Intermediate endophenotypes must be linked to the genotype and must be heritable, and hence must be partially expressed in unaffected relatives. They can indicate liability to the disorder and also provide information regarding potential mechanism of the disorder. Biomarkers do not generally require linkage to the genotype, although they may, as in the example of HER2 genes for breast cancer or the APOE4 gene for Alzheimer's disease. Understanding of the biological link or mechanism of action between a biomarker and a disease state is not necessary for its utility. However, biomarkers may provide clues about underlying pathophysiology through their mechanisms of action and disease‐related derangement thereof. Measurement of MMN within traditional oddball sequences does show acceptable test–retest reliability^40^ that is within the range of other monitored biological indices, such as blood glucose levels in diabetes.^41^

Whether MMN is a biomarker for schizophrenia and which type of biomarker have been contentious for decades. Original work in major mental illnesses showed a large effect size deficit in schizophrenia,^19^ but relatively spared MMN in affective disorder,^42^ suggesting MMN might be a specific biomarker of disease presence (a diagnostic biomarker). Many other studies indicated specificity for schizophrenia versus mania,^43^, ^44^ even at first hospitalization.^45^ Two issues arose that questioned whether MMN was a diagnostic biomarker for schizophrenia. First, the specificity of MMN reduction to schizophrenia was questioned. Several papers reported reduced MMN in affective disorder,^46^, ^47^ suggesting a lack of specificity to schizophrenia, with significant loss in bipolar disorder, although not as severe. Lack of specificity to the disease is critical, as MMN could not be used as a diagnostic biomarker for schizophrenia. Still, it could be useful as a biomarker of disease‐related processes common to the different illnesses in which it was reduced, or serve in other useful biomarker roles. The second issue that suggested MMN was not a diagnostic biomarker for schizophrenia was its apparent lack of reduction at the first hospitalization for psychosis within the schizophrenia spectrum. Salisbury et al.^45^ and Umbricht et al.^48^ both reported a lack of reduction in MMN among first hospitalized individuals. An accurate reading of Umbricht et al.^48^ indicates that first‐episode individuals with some college education had larger MMNs than those that did not attend college, although the group as a whole did not differ significantly from healthy individuals. Several other groups also reported a lack of MMN reduction in first‐episode schizophrenia,^49^ but some groups have reported MMN reductions in first hospitalized schizophrenia‐spectrum individuals.^50^ As reviewed in Salisbury et al.,^51^ studies that included individuals over 1 year from first hospitalization had MMN reductions as large as in long‐term illness, whereas individuals <1 year had more variable results, largely little to no reduction, but including some with sizeable reductions.^52^ Meta‐analyses^2^, ^3^ indicated that MMN reduction at first episode was roughly half as large as in long‐term illness. This was largely driven by studies that used duration‐deviant stimuli (i.e., sounds that were longer than those in the repeated pattern); the effect size for pitch deviant MMN (i.e., elicited to sounds that were higher or lower in frequency) in first‐episode individuals was essentially zero.^3^, ^50^ Interestingly, despite lack of scalp EEG‐measured MMN reductions, source‐localization with EEG^53^, ^54^ suggests subtle reductions may be detected at the generator level. In longitudinal assessment of MMN in the first few years following a first hospitalization, Salisbury et al.^55^ showed that MMN evinced progressive reductions in schizophrenia‐spectrum individuals that correlated with progressive left auditory cortex gray matter reduction, and suggested MMN was a biomarker of disease trajectory or, in the NIH‐FDA nomenclature, a monitoring biomarker. Further, Salisbury et al.^55^ suggested MMN in early disease course could be used as a response biomarker, in that as an outcome measure, interventions that kept MMN large would likely affect the underlying pathophysiology of progressive gray matter loss.

Despite the controversy over whether MMN was, in fact, reduced in first‐episode psychosis (a necessary condition for it to be a biomarker of psychosis presence or risk), reports emerged that suggested it was reduced in individuals in the pre‐psychosis or prodromal state. Brockhaus‐Dumke et al.^56^ reported a slight but nonsignificant reduction of MMN in subjects at risk for conversion to psychosis. Subsequently, the same group compared individuals that transitioned to psychosis to individuals in the at‐risk group that did not, reporting lower MMNs in converters to psychosis, or individuals truly in the prodrome for psychosis.^57^ Among clinical high‐risk (CHR) individuals, only a small percentage actually are prodromal for psychosis. The majority of individuals are not going to develop a psychotic disorder. Thus, logically, broad reductions in MMN, if consistent among all CHR individuals, cannot be used as a risk predictor for incipient psychosis. Demonstrating reductions in only those individuals that transition to psychosis is a critical outcome if MMN is to be considered a risk biomarker. Early studies tended to have relatively larger percentages of converters/transitioners as they tended to select ultrahigh‐risk individuals. Transition rates were as high as 30%–40%.^58^, ^59^ However, these rates were higher than average. In their review of ultrahigh‐risk studies, Simon et al.^60^ reported an overall rate of 24% transition to psychosis, and indicated that the more recent studies showed even lower conversion rates. This is exemplified in recent larger studies of CHR individuals. For example, in a large mega‐analysis of magnetic resonance imaging data in CHR individuals,^61^ 253 out of 1487 CHR individuals followed up transitioned to psychosis (17%). In a recent multicenter study of MMN among CHR individuals, Hamilton et al.^62^ reported that 77/315 (24.4%) of followed‐up individuals transitioned to psychosis. From the same multicenter study, Seidman et al.^63^ reported that 89/689 transitioned to psychosis, that is, only 12.9%. Thus, it is likely that CHR samples comprise 15%–20% of truly prodromal individuals with 80%–85% of individuals not prodromal for psychosis. For MMN to qualify as a susceptibility/risk predictor for incipient psychosis, it must be relatively spared in nontransitioners and selectively reduced in transitioners. Studies that show reductions in CHR individuals as a whole would therefore not support MMN as a susceptibility/risk biomarker for schizophrenia, but instead suggest an association with broad general psychopathology.

As noted above, early studies reported reduced MMN in CHR individuals and, at times, further reduced MMN in individuals that transitioned to psychosis. Shin et al.^64^ reported a reduced right hemisphere MMN dipole moment in 16 ultrahigh‐risk‐for‐psychosis individuals using magnetoencephalography, but did not assess transition. Given the caveat that CHR is only ~20% prodromal for schizophrenia, this suggests a link with general psychopathology. Brockhaus‐Dumke et al.^56^ found no significant reduction in 43 CHR individuals, with reductions of ~0.1 µV. Subsequently, Bodatsch et al.^57^ from the same group reported reduced‐duration MMN in 25 CHR converters to psychosis versus 37 CHR nonconverters, but no differences in pitch MMNs. Importantly, duration MMN in CHR individuals that transitioned to psychosis was smaller than in nonconverters and healthy individuals, while nonconverters were not reduced relative to healthy individuals. These results are consistent with duration MMN being a risk biomarker, and Bodatsch and colleagues suggested transition to psychosis could be predicted by smaller MMNs among CHR individuals. Atkinson et al.^52^ reported reduced‐duration MMN in an ultrahigh‐risk group (*n* = 30), but did not assess transition to psychosis. In a larger longitudinal report, Atkinson et al.^65^ reported that MMN in 80 ultrahigh‐risk individuals was not reduced compared to healthy individuals, and at ~1‐year follow‐up in 67 individuals, only 10% had transitioned to psychosis and were indistinguishable from nonconverters. Shaikh et al.^66^ reported similar findings as Bodatsch et al.^57^ with smaller duration MMN in 41 high‐risk subjects, with 10 converters to psychosis showing smaller MMNs than 31 nonconverters. It was unclear whether nonconverters were statistically smaller than healthy individuals. Recently, Hamilton et al.^62^ reported on MMN to three deviant‐types (pitch, duration, combined pitch and duration) in a large sample of CHR individuals. They reported reduced MMN across all deviant types in 77 CHR converters to psychosis compared to 238 nonconverters (Cohen's *d* = 0.27) and 241 healthy individuals. Analysis of the separate deviant type *z*‐scores at the frontal electrode site *Fz* presented in their eTable 1 with uncorrected *t*‐tests indicated no significant pitch MMN reduction or duration MMN, but a trend‐level reduction for combined pitch and duration deviant (*p* = 0.09) among all CHR individuals versus healthy individuals. Comparison of CHR converters to psychosis versus healthy individuals revealed trend‐level reductions in pitch MMN (*p* = 0.08), and significant reductions in duration MMN (*p* = 0.05) and double deviant MMN (*p* = 0.05). Comparison of CHR converters to psychosis versus nonconverters revealed no significant difference in pitch MMN, but differences in duration MMN (*p* = 0.05) and double deviant MMN (*p* = 0.05). Finally, comparison of CHR nonconverters versus healthy individuals revealed no significant reductions in pitch MMN or duration MMN, but significant differences in double deviant MMN.

Although together these findings suggest that combined pitch and duration MMN could be more sensitive to deficits in truly prodromal CHR individuals,^67^ as noted by the authors, the effect sizes for each type of MMN are too small to be useful as a risk biomarker. Although small reductions in MMN may be detected among CHR individuals, and truly prodromal individuals may have more substantial reductions, the degree of impairment is too small. On balance it appears that smaller MMN may be useful for detecting abnormal mechanisms in disease progression but evidence to date indicates that MMN is unlikely to be useful as a risk biomarker for transition to psychosis.

Although evidence suggests limited potential for MMN as a useful prediction/risk biomarker or diagnostic biomarker, it is nonetheless impaired in a subset of individuals in early course psychosis. Recall that Umbricht et al.^48^ demonstrated smaller MMN among individuals at a first psychotic episode who did not finish high school or attend some college compared with first‐episode individuals that did attend at least some college. This suggests that individuals that were affected earlier, or perhaps had a longer illness duration prior to hospitalization as reflected in poorer social/occupational functioning, had more impaired MMN, presumably reflecting more of the pathology giving rise to reduced MMN in schizophrenia. In the first report of longitudinal assessment of pitch MMN following first break, Salisbury et al.^55^ reported that among individuals at first break, where the group mean did not differ from the healthy individual group mean, those first‐episode schizophrenia individuals with a smaller MMN had reductions in the gray matter of left transverse temporal gyrus (also known as Heschl's gyrus [HG]). This area contains primary auditory cortex, and the gray matter volume of HG correlated with pitch MMN amplitude only among first‐episode schizophrenia individuals, not among healthy individuals or individuals with bipolar disorder with psychotic features. Salisbury et al.^55^ suggested that MMN, then, was an index of the cortical gray matter loss in left temporal lobe observed in early psychosis, putatively tied to a loss of dendritic spines characteristic of the disease.^68^, ^69^ This association between left HG and pitch MMN was replicated in a new sample of first psychosis individuals and also demonstrated for duration MMN.^70^ Furthermore, novel use of the HCP parcellation indicated both overlapping and distinct auditory cortex and inferior frontal auditory‐executive gray matter correlates of individuals MMN amplitude among first episode individuals.^71^ Furthermore, Salisbury et al.^55^ retested individuals on average 1.5 years later. Among first‐episode psychosis individuals, marked pitch MMN reduction was present, and this reduction correlated strongly with progressive gray matter loss in left HG, further supporting the notion of MMN as a monitoring biomarker of disease progression.

A progressive reduction of MMN following first psychotic episode has also been observed by other groups.^72^, ^73^ However, some reported no longitudinal change. For example, Koshiyama et al.^74^ reported reduced‐duration MMN but not pitch MMN at first psychotic break, with no progression of either MMN at the ~2‐year follow‐up, and Higgins et al.^75^ reported reduced MMN in schizophrenia individuals within 3 years of first psychosis that showed slight but nonsignificant progression at the ~1‐year follow‐up. Although the data are not uniformly positive, evincing some degree of disagreement, it appears the weight of evidence suggests duration MMN is only slightly reduced in the prodrome, at best moderately reduced among first‐episode schizophrenia individuals, becomes markedly reduced in the first 5 years of illness, and is severely reduced in longer‐term illness. More empirically, meta‐analyses indicate that an effect size ~1 standard deviation (0.8–1.4) among long‐term illness^1^, ^2^, ^3^ is less than half as large in first‐episode individuals at 0.4 standard deviations, and likely even smaller among CHR individuals (0.2–0.4^2^, ^62^). Thus, MMN does appear to track disease progression, supporting its utility as a monitoring biomarker potentially related to the underlying pathophysiology that leads to reduced gray matter in temporal and frontal cortices in the early stage of the disease process (see^76^, ^77^, ^78^, ^79^, ^80^, ^81^, ^82^ for focal longitudinal gray matter changes).

## WHAT BIOLOGICAL CHANGES CAN BE MONITORED WITH MMN?

Despite potential as a monitoring biomarker, it remains unclear precisely what underlying change is being monitored. MMN is correlated with gray matter changes occurring during early disease course, but the specific mechanism that might link the two is unknown. Links to pharmacologic interventions supporting the potential of MMN as a response biomarker may hint at underlying pathophysiology. Although the neural pathophysiology giving rise to schizophrenia remains a mystery, there is likely a role of dysfunction in synaptic plasticity NMDA receptor,^83^ a slow‐acting glutamate receptor that fires only with coincident fast‐acting glutamate (α‐amino‐3hydroxy‐5methyl‐4‐isoxazolepriopionic acid [AMPA]) receptor activity, and is markedly enhanced via glycine binding as a co‐agonist. The NMDA receptor and its glycine binding site play a crucial role in long‐term potentiation, long‐term depression, and synaptic plasticity. Phencyclidine (PCP) produces the psychotomimetic effects most similar to schizophrenia,^84^, ^85^ and is an NMDA receptor antagonist. Much of the early human and animal pharmacology examined the sensitivity of MMN to drugs that acted at the NMDA receptor. In monkeys, administration of NMDA antagonists leads to reduction of MMN, but no change to the initial sensory ERPs, like N100.^86^ In humans, administration of psychotomimetic drugs that act at the NMDA receptor leads to a reduction of the MMN, and the degree of drug‐induced psychosis correlated with the amount of MMN reduction.^87^ Abnormality of the NMDA receptor is a crucial part of the puzzle of the pathophysiology of schizophrenia. MMN is heavily dependent on NMDA function, vital to the expression of context‐dependent learning. Thus, MMN can provide an objective neurophysiological probe of the current functional state of the NMDA system. As such, MMN should also serve the function of a response biomarker should agents successfully alter the NMDA system in a manner that favors the underlying learning/relevance filtering process.

Antagonists at the NMDA receptor induce an interleukin‐6 anti‐inflammatory response that activates production of superoxide which in turn leads to selective dysfunction of parvalbumin GABA cells.^88^ These fast‐acting interneurons are critical for quickly inhibiting pyramidal excitatory activity (and in local synchrony), and as a consequence of neuroinflammation, the GABA cells may fail to adequately inhibit the pyramidal cells. Theoretically, this overall inhibitory deficit leads to abnormalities in normal synaptic plasticity of the pyramidal cell with eventual regression of dendritic arborization just short of apoptosis, or Glu‐induced dendrotoxicity. Several studies have used MMN recordings alongside treatment trials targeting the glycine receptor. Although the sample was small, including only seven individuals with schizophrenia, Lavoie et al.^89^ reported improvement of MMN with treatment with *n*‐acetyl cysteine, a glutathione precursor, that had anti‐oxidant effects. Greenwood et al.^90^ reported increased‐duration MMN in schizophrenia participants after acute administration of *n*‐acetyl cysteine but this affect was no longer present after 6 weeks of treatment (*n* = 22). Yang et al.^91^ also report a nonsignificant increase in duration MMN after acute administration of *n*‐acetyl cysteine in nine persons with schizophrenia. Retsa et al.^92^ found improvement in MMN amplitude after 6 months of *n*‐acetyl cysteine treatment in a manner that was attributed to increased activity in the left auditory areas (*n* = 15). Kantrowitz et al.^93^ likewise reported increased pitch MMN in schizophrenia with d‐serine treatment, an NMDA glycine receptor agonist.

Another small body of literature suggests MMN may be improved with agonists acting at the α7 acetylcholine receptor. In a small sample of 12 individuals with schizophrenia, Dulude et al.^94^ showed duration MMN improvement with nicotine, and suggested acetylcholine may modulate cortical timing systems. However, in a previous study of eight individuals with schizophrenia, Inami et al.^95^ reported no effects of nicotine in patients. Likewise, Fisher et al.^96^ did not observe increases in duration MMN with nicotine, but rather observed a shortened latency for intensity deviants. A more recent study^97^ reported a dose‐dependent improvement in MMN amplitude in schizophrenia with an α7 partial agonist. In summary, evidence for nicotine agonists improving MMN in schizophrenia is modest, at best. The α7 acetylcholine receptor systems have also been targeted in schizophrenia trials using CDP‐Choline, a supplement with agonist properties. Aidelbaum et al.^98^ observed baseline amplitude‐dependent effects in schizophrenia with clear increases in MMN in those with low baseline MMN, but decreases in those with high baseline amplitudes (subsamples *n* < 10). Kruiper et al.^99^ also assessed acute effects of clonidine in 20 persons with schizophrenia finding nonsignificant (but visible) amplitude increases. For review on nicotine effects on MMN in schizophrenia and healthy individuals, see Smucny and Tregellas.^100^ For review of NMDA receptor modulators and other drugs, including nicotine, supporting MMN's role as a response biomarker of target engagement, see Kantrowitz.^101^

The results of studies on pharmacological interventions are encouraging with respect to the responsiveness to agents targeting the NMDA system. Although it should be noted that most are small samples, the findings suggest MMN is well‐suited as a response biomarker for targeted interventions. It is perhaps not surprising that the findings are complex as they are the results of attempts to modulate context‐dependent learning within a dynamic system. While clinical trials are vital, preclinical research designed to test how and why smaller MMN arises present another promising avenue to deepening understanding of the underlying system and its disruption in schizophrenia.

## CAN WE STUDY THE SAME PROPOSED BIOLOGICAL CHANGES USING MMN IN PRECLINICAL MODELS?

Valid animal models of schizophrenia are a major challenge for science given that the disease affects the highest level of intellectual functioning in humans. Most preclinical models of schizophrenia are based on rodent models with pros and cons for both mouse and rat models. There are fewer nonhuman primate studies. Because of the heterogeneity of the clinical phenotype of schizophrenia, it is an incontrovertible fact that no animal model is capable of recapitulating even a fraction of the phenotypic expression of the disorder. However, animal models of schizophrenia that recapitulate one or more established biomarkers are feasible and potentially very informative. As reviewed earlier, MMN does not meet the criteria as a diagnostic biomarker for schizophrenia but may be useful as a biomarker of disease‐related processes common to different illnesses in which it is reduced to varying degrees. Importantly, with appropriate controls, MMN can be measured across species, including rodents, allowing investigation of the microcircuits contributing to reduced MMN in animal models of schizophrenia^102^ and therefore potentially helping to drive the development of new treatments.

One of the many advantages of using MMN as a biomarker in rodent models is that identical recording, data‐processing, and signal‐extraction methods can be used in human and rodent studies combined with the same oddball stimulus paradigms,^103^ which at face value seem to ensure equivalence of the rodent and human MMN. Furthermore, its elicitation is not reliant on the subject paying active attention to sounds. However, despite these identical elicitation and recording methods, it is not a simple matter to show that the rodent brain is capable of generating an MMN‐like response. Nor has it been a simple matter to demonstrate that certain animal models of schizophrenia reproduce an MMN with reduced amplitude.

To show MMN‐like properties, it is important that a larger response to a deviant sound can be shown to reflect the detection of events that violate a prediction emergent from learning a contextually relevant regularity and not just a less frequently encountered sound property. Given that MMN is extracted as a difference wave between responses to rare and frequent sounds, the main uncertainty is whether differential response suppression of generators of evoked responses to the frequent compared to rare sounds accounts for the MMN difference potential, rather than genuinely reflecting a violation of a predictive rule on the occurrence of a rare stimulus. It was recognized many decades ago by Schroger and his colleagues that special control sequences were required to demonstrate a component that could be attributed to factors other than differential response suppression or stimulus‐specific adaptation (SSA) to the repetitive frequent stimulus.^104^, ^105^ These sequences control for the rarity of the stimulus but in a different context in which either there is no predictive model that the rare stimulus violates because of random presentations of multiple different standards (many standards control sequence) or because the sound is as rare as the oddball but is not unexpected (cascade control sequence^106^). Both types of sequences have confirmed the presence of a prediction error signal in human MMN generation. Further, it has been recently shown that it is the prediction error component of MMN that is reduced in schizophrenia,^107^ consistent with the observation that traditional oddball sequences reveal groups’ differences to be attributable to smaller deviant response amplitudes. Use of comparable sequences in rats has allowed a similar conclusion to be drawn—that is, that the rat brain can produce a prediction error signal, independent of SSA. This is true for both surface recordings^108^ and local field potentials (LFPs) recorded intracortically, and in subcortical recordings along the auditory pathway.^109^ Investigations in nonhuman primates (NHP) have argued that MMN is present but none to date have implemented the appropriate controls that ensure that there is a prediction error component of NHP MMN.^110^, ^111^, ^112^, ^113^ On the other hand, intracranial LFP recordings and single‐neuron activity from cortical and subcortical areas of mouse brain^109^, ^114^, ^115^ have employed appropriate controls to demonstrate evidence of prediction error in these recordings but not yet in surface recordings from the mouse brain as the appropriate studies have not been reported.

A multitude of animal models of schizophrenia have been developed based on known risk factors or suspected causes: drug‐induced models based on accepted models of neurochemical imbalance causes of schizophrenia, neurodevelopmental insults based on the epidemiology of schizophrenia or genetic predisposition informed by multiplex family and genome‐wide association studies.^116^, ^117^ If the implemented model results in the schizophrenia endophenotype of reduced MMN, one can then proceed to investigate the neural basis of the MMN reduction. An assessment of the outcomes from application of at least two of these models, however, has had limited success in advancing knowledge of the basis of MMN reduction in schizophrenia. In terms of drug‐induced animal models, the role of NMDA‐receptor function in MMN generation in rodents^118^, ^119^ has confirmed human research that administration of glutamate NMDA receptor antagonists reduces MMN^87^ consistent with an NMDA receptor hypofunction model of schizophrenia, although the precise microcircuit mechanisms underlying this reduction are still largely unknown.^120^ It has been much more difficult to demonstrate that another class of animal models based on neurodevelopmental insults that increase the risk of schizophrenia result in reduced MMN. However, our recent research^121^ has demonstrated that adult rat MMN amplitude is reduced in a model of schizophrenia based on maternal immune activation (precipitated by maternal infection for example) combined with chronic adolescent cannabis use but only in a certain oddball context, namely in sequences in which the probability of the deviant varied and only in male rats. No MMN reduction was observed in female rats. Probability of the deviant (or rather its inverse, the probability of the standard) affects the precision of the predictive model, suggesting that impaired precision‐weighting of MMN in the treated animals could account for the effects. Hence, reduced MMN in such animal models may be very dependent on the oddball context used to elicit MMN. This is an important caution for the design of future animal studies to determine the microcircuit mechanisms underpinning reduced MMN in these models.

It should be noted that much can be learnt about the mechanisms underlying reduced MMN from rodent studies even in the absence of an animal model of schizophrenia exhibiting MMN changes. In particular, microcircuit functioning of pyramidal cells and GABAergic inhibitory interneurons, parvalbumin and somatostatin positive interneurons within cortical layers (layer 2/3 in particular) of the mouse brain has attracted considerable attention. There is some evidence that each of these three cell types exhibits a prediction error signal in their response to a deviant stimulus,^114^, ^115^ but there seems to be an emerging consensus from rodent studies that suppression of somatostatin interneurons (but not parvalbumin) plays a major role in prediction error response of pyramidal cells.^115^, ^120^ In terms of understanding reduced MMN in schizophrenia, this is an exciting finding as there is evidence from post‐mortem studies of schizophrenia brain tissue of reduced somatostatin activity.^122^, ^123^ Multiple investigations of the rodent brain have involved a variety of methods: electrophysiological recordings of individual neurons, two‐photon imaging, optogenetics and transgenic mouse lines, but when combined with appropriate controls in the design of stimulus sequences,^108^, ^109^, ^114^, ^115^, ^120^ the power of animal models in understanding fundamental mechanisms in the generation the prediction error component of MMN is apparent. These are early days in research on animal models but the potential of these approaches not only in understanding cellular mechanisms leading to reduced MMN in schizophrenia but also for pointing the way to novel treatments is very promising.

## DO METHODS MATTER TO WHAT WE LEARN ABOUT MMN IN SCHIZOPHRENIA?

Throughout this review, there have been various references to MMN effects that might depend on the experimental methodology (e.g., a specificity to duration or pitch MMN^124^). At face value, it may seem odd for the findings derived from such simple sequence structures to depend on tonal properties and the reasons for this are not well understood. Nonetheless, speculation as to why is aided by increasing appreciation of the network of brain regions that contribute to the process of using recent experience to best estimate the likely stimulation to follow. Because the source location of auditory activation is deviant‐specific,^125^ different sound deviations could be differently sensitive to underlying vulnerabilities based on place of encoding or complexity of encoding. For example, differences in the findings associated with pitch and duration MMN have been hypothesized to relate to the more complex way sound duration is encoded relative to the comparative simplicity of place‐coded topographical representation of pitch.^126^, ^127^, ^128^ An additional difference between a duration and pitch deviant is that the former matches the regularity before it deviates (i.e., it is initially identical to the repeating sound until it extends longer or ceases earlier than it should) while the latter deviates immediately upon sound onset. This delayed point of deviance is certainly one factor that shifts the time point at which the MMN appears such that the additional negativity evident at the scalp overlays later components of the auditory response (e.g., P2 and N2, rather than N1) and different time‐frequency dynamics. Furthermore, a duration deviant sound is technically always a double deviant because at the short durations typically used, a change in sound duration will not only sound shorter or longer but indeed softer or louder, respectively, due to a summation of energy over time.^23^, ^129^, ^130^ While it is not exactly known why group differences are sometimes better indexed by a change in duration, this double‐deviance might be the best explanation given more recent observations that the combination of the two (a sound that is different in both pitch and duration from the regular sound) might be an even better group discriminator.^67^

Although tempting to assume an auditory cortical pathology to explain smaller MMN in schizophrenia, such as cellular mechanisms reviewed earlier, the network servicing auditory inferences engages several brain areas inclusive of the prefrontal cortex.^131^, ^132^, ^133^ Indeed, animal studies suggest that the sensitization to pattern deviations is most pronounced in these more rostral regions of the network, and recent approaches to data analysis enable attributions to be made to different sources along the auditory pathway.^35^, ^36^, ^133^, ^134^, ^135^ Moreover, different brain regions are purported to extract regularity information over different temporal intervals such that shorter timescale information that can be gleaned by sensory areas is modulated by longer timescale information supported by more rostral areas, such as the prefrontal cortex.^136^, ^137^, ^138^ Patterns that are only evident over longer timescales can therefore theoretically be used to weight the dependence of MMN on higher areas of the cortical network.^139^, ^140^, ^141^, ^142^ Finally, theoretical perspectives on the varied reasons for different MMN amplitudes have also progressed. What was once considered probabilistic inference based on sensory memory^5^ has been augmented by evidence of statistical learning of transitional probability^143^ and even speculation that the system is engaged in hierarchical inference.^139^, ^140^, ^141^, ^142^, ^144^ The latter may be of particular interest to understanding smaller MMN as it encourages a consideration of when a system might settle with a “good‐enough” model of what is likely in an environment versus continuing to reduce the disruptive effect of model mismatches based on when sound is perceived to carry information of value to continued learning. In summary, methods do matter with respect to what we can learn about altered MMN in schizophrenia. These theoretical and methodological developments continue to enrich the depth and breadth of questions that can be brought to bear on what is altered in schizophrenia that is evidenced in changed auditory responsiveness and whether it can be treated or prevented.

## CONCLUSIONS

This review has considered what we have learned about schizophrenia by recording the MMN component of the auditory event‐related potential. While evidence to date might suggest that MMN lacks the specificity and effect sizes required for practical use as a biomarker for diagnosis and prognosis, MMN continues to exhibit potential as a monitoring and response biomarker. Furthermore, it is evident that the field has advanced (and continues to advance) through a combination of clinical studies, a deeper understanding of factors that influence MMN size, preclinical research in animal models to test treatment pathways and underlying mechanisms, and sophisticated analytic techniques. Concurrent advances in understanding the pathophysiology of schizophrenia are also likely to further inform what it is that MMN indexes about functional change. For example, the ability to use stem cells from patients to derive neuronal cultures (a technique called induced pluripotent stem cells) enables a more direct exploration of how a patient's genetic background affects differences in neuronal development. As reviewed in Howes and Onwordi,^25^ such studies are yielding new insights into how changes in gray matter volume might arise, even linking this to early life events affecting subsequent patterns of vulnerability in neurodevelopment. Whether these or future advances change the way we use MMN remains to be seen but the obligation to pursue its potential remains strong with the field of research expanding in its multidisciplinarity and its promise to deliver on better knowledge and treatment for schizophrenia.

## AUTHOR CONTRIBUTION

All authors contributed to the original draft and final editing of this review.

## CONFLICT OF INTEREST STATEMENT

The authors declare no conflicts of interest.

## ETHICS APPROVAL STATEMENT

N/A.

## PATIENT CONSENT STATEMENT

N/A.

## CLINICAL TRIAL REGISTRATION

N/A.

## Data Availability

N/A.
